# Mr. Ring, in Reply to Dr. Kinglake

**Published:** 1806-11

**Authors:** John Ring


### To the Editors of the Medical and Phyſical Journal.

Gentlemen,

It appears by the last Number of your Journal, that Dr. Kinglake is one of those authors, who think my remarks on the subject of vaccination too severe; a circumstance of which those are the best judges, who know all the wanton, and petulant, and unmerited attacks, to which the writers on the subject of vaccination have been continually exposed; partly from persons who are interested in opposing the practice, and partly from persons who are ignorant of what has been written, but anxious to bring their names before the public, on every occasion.

When delivering his opinion on my conduct, he pretends to be impartial, but with an ill grace; when he has commenced a controversy with me, when he confesses the force of my satire, and seems still to smart under the lash. He allows some weight to the evidence which I have adduced in favour of my sentiments; but is still inclined to entertain his own, till he himself has investigated the origin of the cow-pock; which, every one must acknowledge, he has an undoubted right to do. He is, however, a little too hasty in thinking, that I regard any opinion of mine as a *non tangendum*. I claim no exemption in that respect, nor any privilege, which I am not willing to allow to Dr. Kinglake, or any other author. But though I am not troubled with a *noli me tangere*, that is no reason why I should suffer Dr. Kinglake, or any man, to pull me by the nose with impunity.

Dr. Kinglake contends, that the public in general, and even medical practitioners, still continue sceptical on this point, which is no wonder, when we consider how little some of them are acquainted with what has been written on the subject. I am happy to hear that Dr. Kinglake has adopted vaccination in his own family; and am far from wishing to prevent him from instituting a series of experiments, in order to ascertain the source of the disease, if he thinks it is not already ascertained.

In one part of Dr. Kinglake's reply, there is a misconstruction of my meaning; which must not be suffered to pass uncorrected. When he says, he did not charge me with using the phrase indisputably safe and efficacious, he says what is perfectly correct; but when he says, I represent the insertion of acrid and fœtid matter from the diseased heels of a horse, as indisputably safe and efficaious, he is not correct, correct, nor warranted in that assertion. No one can entertain a greater dread and abhorrence of such a practice than I do.

Even when matter is taken from the cow-pock itself, whether in the brute animal or the human subject, if I represent it as safe and efficacious, provided it be taken from the genuine pock, at an early stage, I by no means represent or admit, that the acrid and fœtid matter which issues from the sore nipples of cows, or the sore arms of children, who have had the cow-pox, is also safe and efficacious. I said, indeed, that Dr. De Carro, and Dr. Friese, now inoculate indiscriminately with equine and vaccine matter; but had no reason to apprehend, that any one would suppose I meant any other equine matter, than what I just before specified, as received by them from Dr. Sacco.

It must be recollected, that I was not then alluding to the safety, but the activity and efficacy of the matter; and maintaining that what was transmitted by Dr. Sacco to Dr. De Carro was of the same nature with the vaccine virus. It is therefore a great misrepresentation, and a perversion of terms to assert, that I have on any occasion represented every kind of matter, either from the horse or the cow, as either efficacious or safe. Common sense revolts at such an idea; and I cannot imagine how Dr. Kinglake could suppose it was ever entertained, either by me, or any other man. So far from thinking it safe, I have, on many occasions, described the ill consequences of inoculating with putrid matter, whether from the brute animal, or the human subject.

Dr. Kinglake thinks me not likely to succeed, in obtaining *rational converts*. He allows that my opinion is highly corroborated by several instances which I have cited; but thinks the modifications and stages of the disorder are not sufficiently pointed out. Here I am once more compelled to observe, that none are so blind as those who *will not see*. In the Journal for August I remarked, that *the virus is most active at the commencement of the disease, before it has acquired the consistence of pus*; and that Dr. Jenner seems to have been perfectly right in his conjecture, *that it is the thin fluid, of a darkish colour, oozing from the recent cracks in the heels of a horse, which communicates the disease in question.*

I also stated the opinion of Sir Christopher Pegge, that the cow-pox proceeds from *the scratchy heel;* which is *widely different from the common grease.* In the Journal for for September I stated, that Dr. Loy inoculated a cow with *limpid matter of grease,* and afterwards, that he inoculated three other cows with *the same lymph,* and succeeded in every instance. I also stated, that he was of opinion, there are two sorts of grease.

In the account of Dr. La Font's experiments, I stated, that the virus was *limpid,* but *rather yellow and filamentous.* This also proved successful; but, as far as can be judged from the effects, though differing in colour, may be considered as only a modification of the same fluid; in consequence of its being secreted from the leg, instead of the heel.

In my Treatise, p. 237, I copied from this Journal, the following extract of a letter from Mr. Grose of Winslow. "I have had many opportunities of conversing with respectable farmers, whose cows were affected with the disease; and they unanimously agree in ascribing it to a complaint in the horse's heel; which is called, from its singularity of making the hair erect, *a scratchy heel.*"

From the preceding paragraphs and others, it must appear evident to every unprejudiced reader, that this experiment is *not still in its infancy,* as Dr. Kinglake pretends; that the labour bestowed on it has *not been fruitless;* and that something precise *has been ascertained,* as to the particular mode of grease, and the period when it exerts its specific infection. If this, or any other matter of grease, at one time 'has no effect,' and at another 'produces its specific influence,' the same may be said of all the matters in the world; and if Dr. Kinglake is determined not to accede to my opinion, till equine or vaccine matter always produces an effect, and a specific influence, I shall never be able to add him to the number of *rational converts.*

He thinks, that if my opinion is well-founded, either the research is extremely difficult, or has not been conducted in a manner calculated to insure success. The only difficulty that I know in this research, is that of finding the proper species of grease, at the proper period: this is what I must leave for those who have more leisure for the task, or more inclination to undertake it.

Dr. Kinglake allows some weight to the arguments drawn from cases of accidental infection: but he says, "*unless the hint is taken up, and carried to demonstrative proof, by a regular series of unequivocal experiments,* it has no title to be received as a philosophical axiom;" nor to be regarded with the confidence, with which it has been regarded regarded by me. Here we are at issue; and I rest my claim to the confidence of the public, on the evidence which I have adduced in this Journal; thinking that the hint *has been taken up, and carried to demonstrative proof, by as regular a series of experiments,* as ever was instituted, in England, in Italy, and in Greece.

Dr. Kinglake pledges himself to undertake the task, of endeavouring to confirm, or refute this opinion; and of course, it cannot long remain undecided. He promises a large store of information to the public in general; and to me in particular, who am sensible that in many respects, I stand in need of all the information which it is in his power to communicate. Should he deign to shed but one ray of light, to guide me through the labyrinth of errors in which I am involved, I shall thankfully receive it.

He thinks my writings voluminous; it is therefore necessary to inform him, that they would have been more concise, and not have been extended to half their present bulk, had not certain sceptics, who had no knowledge of the subject, nor any motive for writing on it, but the vanity of appearing in print, continually provoked fresh discussion.

He thinks the farrier and carter, his colleagues in this inquiry, more admissible authorities than me, because I have not been used to dress horses' heels; and have not had so much insight into the nature of grease. This is no small mortification to me, and no small disappointment to my ambitious and aspiring hopes; not to be enrolled with those worthies in the records of science; not to be inserted with them in the greasy heels of Pegasus, "and shine among the gods."

It is, however, some consolation, under the pressure of this calamity, that although I am not to be admitted as a witness on this occasion, I may perhaps be admitted as a judge, in preference to Dr. Kinglake, or either of his colleagues, because I am better acquainted with the evidence on both sides of the question.

By referring to the analysis of Dr. Sacco's publication, in the seventh volume of this Journal, it will be seen, that he was, for some time, just as confident of his having proved a negative, and disproved Dr. Jenner's theory concerning the origin of the cow-pox, as Dr. Kinglake at present is; yet the documents which I have laid before your readers, furnish abundant proofs, that he is now become one of the most decided converts to this opinion. In the work alluded to, he says, "*It appears to be a rash*

*assertion*

*assertion of Dr. Jenner, that the grease is the exciting cause of the disease in cows, which is called the cow-pox. I have proved the falsity of this opinion, by direct experiments and observations; and have since been informed, that Pearson, Simmons and Woodville, likewise entertain sentiments on this subject, different from those of Jenner."* Dr. Sacco is now converted to Dr. Jenner's opinion, "*by taking up the hint, and carrying it to demonstrative proof, by a regulnr series of unequivocal experiments."* He is now convinced, therefore, that it is not quite so easy to prove a negative as he once supposed; and as Dr. Kinglake still supposes.

After the "sure and solemn pledge of fidelity," and "implicit confidence in vaccine inoculation," which he has given, by "subjecting his infant children to its benign influence," I cannot but regret that Dr. Kinglake still entertains any doubts concerning the origin of the disease; since a difference of opinion on this point, among the friends of the practice, affords a pretext, though a very shabby and pitiful one, for the perverseness and obstinacy of its opponents; who strenuously contend, that if Dr. Jenner is mistaken concerning the origin of the cow-pock, he is also likely to be mistaken concerning its effect.

As Dr. Kinglake deems the inquiry into the origin of this affection of so much importance, I am happy to learn that he has imparted its benign influence to his own family; and trust he will exert *all his benign influence* with his neighbours, and the public; in order to promote the practice, and diffuse that blessing. It is now more than eight years since the time of its promulgation; and I cannot but think, that every human being, who now falls a victim to the small-pox, is a greater *opprobrium medicorum* than the gout; to which Dr. Kinglake is such a sworn enemy, and which he has often been so happy as *to repel.* Thinking, therefore, as I do, that he will soon kill or cure all his patients who labour under this disorder, and repel that enemy from the land, I hope he will turn his victorious arms against another; which is a much greater bane of human happiness, and exercises its ravages on the innocent part of the creation. This will be a more honourable employment, than doing what a farrier can do as well; and a more useful employment, than doing what is already done:

*O curas hominum! quantum est in rebus inane!*

Dr. Kinglake thinks my writings voluminous; but if he had had ever perused them, he would have found, that in proportion to their contents, they are not more voluminous than his own. Volumes are necessary, when we have a dull scholar to teach; but a letter in a Journal is too much, when we write on a topic which we do not understand.

Dr. Kinglake feels hurt at the word *ignorant*; but no word is more applicable to an author, who betrays a want of knowledge in what he pretends to know. I myself was ignorant, till I read Dr. Kinglake's philippic against me; ignorant that I had offended him; ignorant that he was going to write about grease; and ignorant that there was any man living who knew so little of the subject.

He considers my remarks as declamation; but if he will read them once more, and not only read but understand them, he will find that they contain more stubborn facts, than all that he has ever written, since he was able to hold a pen. He talks of *confirming* my authorities, but they require no confirmation; and of *shaking* them, but it is not in his power to shake them. They are fixed on a solid basis; and cannot be shaken by any arguments which he is able to advance.

---

I now come to the second part of Dr. Kinglake's Memoir; which, to use his own phraseology, "was not wanting to prove," that he sometimes ventures to write on a subject which he does not understand. He there lays before his readers the information which he has lately received from two persons concerned in dairies, tending to prove, that the cow-pox generally occurs in cows after the first cast; owing to the inflammation occasioned by milking, before they are accustomed to the process. In this case, he tells us, *pustular* eruptions, appear, containing *a thin transparent fluid!* He also tells us, that when these *vesicles* break, they leave *open sores*; and that these *open sores* are *troublesome to cows in milking*.

This very extraordinary information, like that which was the subject of his former essay, will no doubt excite great surprise; and we shall have a number of sceptics who will never be convinced, till they have instituted a course of experiments with their own hands, and witnessed the miracle with their own eyes: but Dr. Kinglake is "assured" it is true, by "two persons who are extensively concerned in the management of dairies, and very intelligent in their dairy occupations."

These persons, he tells us, *naturally* ascribe the disease to the violence done the teats, in first handling them, in the the act of milking. If this be a fact, he thinks it may in some measure be owing to the process of lactescence disposing the parts to inflammation; an opinion in which most people will agree with him. He confirms this position by analogy; but as the subject is neither new nor abstruse, perhaps any further confirmation of it may be dispensed with.

One of the persons alluded to, assured him, that her cows repeatedly had this complaint, though milked only by females, who had no communication with grease; and that these females, and the cows which they milked, became infected with the same disorder.

This, however, is no more than what occasionally happens in the spurious cow-pox, as Dr. Jenner informed us long ago. Dr. Kinglake however observes, that if the cow-pock arises from this cause also, it does not always arise from the grease. This is true; but it is equally true, that if this was the spurious sort of cow-pox, it was not the genuine sort; and we have reason to suppose it was the spurious sort, since Dr. Kinglake has not brought forward one tittle of evidence to prove the contrary.

This want of evidence is, nevertheless, abundantly supplied by vain conjectures and idle hypotheses; in which I have no inclination to follow him. He thinks the occasional failure of the cow-pock inoculation is owing to the use of a wrong matter; but as far as we can judge from his present effort, it will be a long while before he will find out the right. He allows the merits of Dr. Jenner, and honours me with the title of his elaborate expositor; but thinks it necessary to ascertain the origin of the disease, with much more precision than we have done. This he pledges himself to attempt; and seems confident of success, by the help of the farrier and the carter; and the twin stars in *the milky way*, who, according to his account, have lately thrown so much light on this obscure subject.

Quid dignum tanto feret hic promissor hiatu?

I have now before me, a letter from Dr. Gumprecht, of Hamburgh, in which he says, "all German physicians, and medical authors, are now of your opinion, although they opposed it so much at first; that the cow-pock originates from the horse, and not from the cow."

Dr. Kinglake, however, gives us to understand, that his doubts are still unsatisfied; but I cannot see any reason for his construing my refusal to undertake the task of inoculating inoculating with grease at his request, as an avowal of indifference.

The truth is, that if I submitted to such an idle task, after the evidence which I have adduced, I should rather deserve his pity than his respect. I am well aware, that the remark of a late author is too well founded; and "that it is very seldom a controversial writer will acknowledge he is convinced of his error." All that I can do, is to bring forward rational evidence; and such as I think, ought to satisfy every reasonable man; I know the world too well, to suppose that any evidence which I can bring forward, will satisfy every man. It is well known, that we have sceptics, *who doubt, or pretend to doubt, every thing*; and it is generally allowed, that if any thing can be more absurd and unreasonable than the dogmatist, who is *sure of every thing*, it is the sceptic, who *believes nothing*.

---

In the Review of Dr. Willan's publication, in the last number of this Journal, a remark occurs, which, if not noticed, must naturally lead to an erroneous conclusion. A doubt is there expressed, whether my statement be correct. In Dr. Willan's publication, the doubt is, whether *Mr. J. Ring's* statement be correct. The fact is, that the case neither fell under the observation of Mr. J. Ring nor myself; but of Mr. Powell, then residing at Chatham Barracks. The result of the inquiry made by Mr. J. Ring, at my request, was, that the pustules began to die away on the eighth or ninth day. This is certainly a very equivocal proof of its not being efficient: but probably as unequivocal as any that can be brought forward, of the disorder which ensued being the small-pox. Such as it was, I thought it my duty to record it. I agree with the Reviewer, that many have been safe, in whom the progress has not been slower than the above; and, that if the only consequences to be apprehended are, that mild small-pox which in some instances has occurred after vaccination, it is of little importance to the patient.

I also agree with him, that the subject of *irregular vesicles*, of a *cellular structure*, still requires elucidation. I am not yet convinced of their existence, except in those persons who have had the small-pox, or the cow-pock. I have seen several instances of the kind in those who have had the small-pox before. They are smaller than in those who have not previously had the small-pox, and not so round. They are earlier in their appearance, and more rapid in their course. Sometimes they yield genuine virus, rus, when opened on an early day; but when matter for inoculation has been taken from them at a later period, they have occasionally produced a spurious disease.

---

Dr. Willan's testimony in favour of vaccination cannot but have great weight with the public in general, and the medical profession in particular. He is of opinion, that if real failures occur at all, they are extremely rare. He is also of opinion, that the cow-pock produces no other disease. He tells us, he has carefully examined various cases of eruptions, which were attributed to vaccination, with different physicians and surgeons; and, instead of diseases originating in quadrupeds, constantly found them such as were well known, and fully described by medical authors, above a thousand years ago.

Some people imagine, that if the cow-pock does not excite new cutaneous disorders, it increases the number of the old, and renders them more obstinate. This opinion also Dr. Willan informs us, his own experience would authorise him to contradict; but he thinks he shall refute it in a still more satisfactory manner, by exhibiting lists of the patients at the Carey-street Dispensary, for the years 1797, 1798, 1804, and 1805, communicated by Dr. Bateman; from which it is evident, that the proportion of such eruptions is not increased, since the commencement of vaccination.

While we are thus confirmed in our former opinion, that vaccination is innocent of the mischief laid to its charge by ignorant, self-interested, and ill-designing men, we have the further consolation to reflect, that it has proved serviceable in many inveterate complaints; and especially those of a cutaneous and scrophulous kind. In asserting this well-founded claim of that benign practice, I am far from maintaining, or insinuating, that it is a prophylactic against any future complaint whatever, except the small-pox. On the contrary, I am more and more convinced of the truth of what I have already stated in this Journal and elsewhere, that in this respect, some of the friends of the practice have been too sanguine in their expectations.

This error, however, might have been considered as venial, since it must have proceeded from a zeal in the cause of humanity; but it has been thought a fit object for raillery and derision. One writer, who was a long time unwilling to admit the merits of vaccination, and illiberal in his remarks on that subject, not content with casting aspersions on those enthusiasts who cherished this delusive hope, thinks proper to brand the friends of the practice in general as a set of ideots, for a blunder which they have not committed. He accuses the Vaccinators of boasting, that the cow-pock is an antidote against the plague and other diseases, as well as the small-pox; and heartily laughs at them for this imaginary blunder. Risu inepto res ineptior est nulla.

There are visionaries, it must be confessed, in this, as well as every other scientific pursuit; but if a few of those persons who are engaged in such a beneficial practice, and who daily witness its wonderful effects, are for a time transported beyond the bounds of reason; if they fancy that they have discovered a prophylactic against more diseases than one, that humanity, which inspired the hope, should atone for the transgression. If they were such enthusiasts, as to flatter themselves that they had discovered a panacea, surely they deserved a better return. If they are not entitled to gratitude and esteem, surely they were entitled to a pardon for their mistake; and were rather objects of clemency and compassion, than of ridicule and contempt.

But even if it had been otherwise, and if these enthusiasts had merited the treatment which they have met with, justice required, at least, that a due discrimination should be made between them and others, who were less enthusiastic; and that no one should laugh at them, but those who have never exhibited an equal specimen of credulity. Above all, the Editor of the work in question, should not laugh at others, on account of *their* credulity, having lost a lucrative situation in consequence of *his own*.

These remarks would have been spared, had the friends of vaccination met with the return due to their labours, and been treated with that candour, of which they hear so much, and experience so little; but this is reserved for the opponents of the practice. When the friends of vaccination heard of the misfortune of that gentleman, who now treats them with so much contempt, they did not laugh at him for his credulity; they only pitied his weakness, and lamented his folly.

But, after all, perhaps they are rather unreasonable, in expecting to be exempt from that obloquy which an unwearied and successful perseverance in any great enterprise has never failed to excite. They ought always to remember the lesson, which one of their most virulent opponents ponents bequeathed them,—that envy and jealousy are the inseparable attendants of merit; and they must no more expect to escape calumny and detraction than other men. I am, &c.

JOHN RING.

